# Satisfaction with simulation-based education among Bachelor of Midwifery students in public universities and colleges in Harar and Dire Dawa cities, Ethiopia

**DOI:** 10.18332/ejm/113132

**Published:** 2019-10-31

**Authors:** Arif H. Jamie, Abdusemed A. Mohammed

**Affiliations:** 1 Department of Pediatrics Nursing, Harar Health Science College, Harar, Ethiopia; 2 Department of Midwifery, Harar Health Science College, Harar, Ethiopia

**Keywords:** midwifery, simulation-based learning, learner satisfaction

## Abstract

**INTRODUCTION:**

Simulation-based education using low to high fidelity techniques are common in midwifery professionals’ education, and it is found to be an excellent alternative to fill the gaps in skills teaching and learning. The aim of this study was to assess the students’ satisfaction with simulation-based education and associated factors.

**METHODS:**

A cross-sectional study in academic settings was conducted from March to May 2018. The data were collected through a self-administered questionnaire. Bivariate and multivariate logistic regressions were used to identify factors associated with student satisfaction with simulation-based education and the degree of association was measured by using odds ratios with 95% confidence interval.

**RESULTS:**

Students who were assisted by their teachers during skills demonstration and practice were 5.6 times more satisfied than those who were not assisted (adjusted odds ratio, AOR=5.62; 95% CI: 2.36–13.40, p<0.001). The students who perceived that the way their teachers conducted the skills demonstration was suitable to their learning styles were 22.4 times more satisfied with the simulation-based education (AOR=22.4; 95% CI: 10.8–37.5, p<0.023). Students who perceived that the number of skills practices per semester was enough were 2.3 times more likely to be satisfied with simulation-based education (AOR=2.3; 95% CI: 1.0–5.3, p<0.042).

**CONCLUSIONS:**

The efforts of teachers in assisting their students during skills demonstration, the suitability of the way of teaching to the learning styles of students, and the number of scheduled programs per semester for skills practising were statistically significant factors with satisfaction in simulation-based education.

## INTRODUCTION

The satisfaction of the students determines the ability to learn and practice clinical skills in a controlled environment before they are required to practice on patients, and thus student satisfaction is a central element in midwifery education as it determines the interaction of teaching-learning processes and the applicability of simulate-based learning methods to improve the outcomes of learning^1-3^.

High-quality patient care is only feasible if midwives have received high-quality teaching during their course of study and working years. Current education methods related to the theoretical and practical training of midwives can help them to recognize and manage better patients’ needs by means of simulated cases^4,5^.

Simulation-based education can create a specific learning environment to ensure midwifery students manage their experiential learning by reinforcing clinical skills through different levels of competency. In both undergraduate teaching and post-registration education, simulation is increasingly being used as a teaching tool within midwifery to teach both emergency situations and practice skills^5,6^.

In the traditional instructional methods, teachers provide knowledge to students by lecturing using slides and setting assignments, and so basically teaching the theory; however, currently simulation delivers knowledge by facilitating students to develop the relevant skills^7,8^.

The importance of transferring knowledge and skills, from theory to practice, is vital in establishing clinical credibility. The benefits of simulation are many, not least the fact that such activities can support students in skills acquisition without exposing patients to risk and therefore potentially improving safety. The level of control that can be exerted over the simulation environment is higher compared with ‘real’ clinical situations^9-11^.

In developing countries like those of Africa, accessibility of high-quality technologies is limited and only a few teaching institutions have them; in some situations, old tools of simulation are being used with poorly functional instruments and less interactive methods of teaching and learning^12^. Therefore, the aim of the present study was to assess student satisfaction with simulation-based education and the associated factors, among Bachelor of Midwifery students in public universities and colleges in Harar and Dire Dawa cities, Ethiopia.

## METHODS

### Study setting and participants

The cross-sectional study was conducted from 1 March to 5 May 2018 at Harar Health Sciences College, Haramaya University and at Dire Dawa University. The cities of Harar and Dire Dawa are located in the far-east of Ethiopia, about 514 km and 452 km from the capital city of Addis Ababa, respectively.

A sample size of 243 was calculated, by using a single population proportion formula for a 54.2% student satisfaction taken from a study conducted at the University of Gondar, Ethiopia, and a 10% non-response rate. Ethical clearance was obtained from the Institutional Research Review Board of the College of Health Sciences of Mekelle University (Ref.no.MU-109/2018). Consent was obtained from the administrative bodies of the colleges and from the participants.

### Data collection tools

A structured and pre-tested questionnaire was used for data collection, adopted from the National League for Nursing, which contained four parts: sociodemographic variables, academic demand, level of student satisfaction with simulation-based education, and experience with simulation-based education. The questionnaire was prepared to measure students’ satisfaction with simulation. Participants rated their level of agreement with each item on a 5-point Likert scale (1 = strongly disagree to 5 = strongly agree). The questionnaire was self-administered.

### Data analysis

After data collection, the questionnaire was checked for completeness and coded. The data were entered into Epi-info version 3.5.3 and exported, cleaned and analyzed by using SPSS version-20. Descriptive analyses were performed and bivariate analyses were used to find out the association of independent variables. Variables with a p<0.05 in the bivariate analysis were entered into multiple logistic regression and variables with p<0.05 in the multivariate analysis were considered to have statistically significant associations.

## RESULTS

In all, 241 midwifery students participated in the study with a response rate of 98.8%, the majority studying at the Haramaya (39.4%) and Dire Dawa universities (39.4%). The study participants were predominately male (55.6%), under 25 years of age (92.5%) with a mean age of 22.1 ± 1.6 years, in their 3rd (44.4%) or 4th (55.6%) year of the course. Among the participants, 124 (51.5%) scored a cumulative grade point average (CGPA) of higher than the mean (3.15), while 129 (53.5%) of the participants said that the Department of Midwifery was their first option of study, and 174 (72.2%) were interested in joining and studying midwifery, but 50.2% reported the need of assistance with their ability to understand English.

### Experiences of midwifery students with simulation-based education

The level of students’ satisfaction with simulation-based education was also assessed; 171 (70.95%) reported that they agree and strongly agree that they are satisfied with the educational curriculum. Table 1 provides the responses of the participants to specific evaluation domains.

**Table 1:** Experiences of students with simulationbased education and its associated factors among undergraduate midwifery students in public universities and colleges in Harar and Dire Dawa cities, Eastern Ethiopia, 2018

*Variables*	*Categories*	*Satisfied n (%)*	*Unsatisfied n (%)*
My teacher provides me accurate information about skills requirements	Strongly disagree	9 (3.7)	5 (2.1)
	Disagree	42 (17.4)	40 (16.6)
	Neutral	94 (39.0)	21 (8.7)
	Agree	26 (10.8)	4 (1.7)
	Strongly agree	0	0
My teacher explains learning objectives for simulation-learning at the beginning of the period	Strongly disagree	3 (1.2)	3 (1.2)
	Disagree	49 (20.3)	56 (23.2)
	Neutral	72 (29.9)	6 (2.5)
	Agree	38 (15.8)	4 (1.7)
	Strongly agree	9 (3.7)	1 (0.4)
During skills demonstration, my teacher gives me enough time to meet the objective(s)	Strongly disagree	6 (2.5)	10 (4.1)
	Disagree	77 (32.0)	49 (20.3)
	Neutral	74 (30.7)	10 (4.1)
	Agree	12 (5.0)	0 (0.0)
	Strongly agree	2 (0.8)	1 (0.4)
During skills demonstration, my teacher assists me in developing long-term skills	Strongly disagree	5 (2.1)	14 (5.8)
	Disagree	28 (11.6)	33 (13.7)
	Neutral	94 (39.0)	14 (5.8)
	Agree	40 (16.6)	8 (3.3)
	Strongly agree	4(1.7)	1 (0.4)
During skills demonstration, students’ different backgrounds are taken into account	Strongly disagree	1 (0.4)	12 (5.0)
	Disagree	31 (12.9)	46 (19.1)
	Neutral	100 (41.5)	5 (2.1)
	Agree	37 (15.4)	7 (2.9)
	Strongly agree	2 (0.8)	0 (0.0)
During skills demonstration, the way my teachers taught the simulation is suitable to the way I learn	Strongly disagree	3 (1.2)	15 (6.2)
	Disagree	36 (14.9)	48 (19.9)
	Neutral	99 (41.1)	7 (2.9)
	Agree	15 (6.2)	8 (3.3)
	Strongly agree	7 (2.9)	3 (1.2)
The teaching methods used in the simulation are helpful and effective	Strongly disagree	2 (0.8)	13 (5.4)
	Disagree	37 (15.4)	47 (19.5)
	Neutral	94 (39.0)	4 (1.7)
	Agree	38 (15.8)	6 (2.5)
	Strongly agree	0 (0.0)	0 (0.0)
There are enough skills practising programs per semester	Strongly disagree	3 (1.2)	12 (5.0)
	Disagree	41 (17.0)	26 (10.8)
	Neutral	117 (48.5)	28 (11.6)
	Agree	9 (3.7)	4 (1.7)
	Strongly agree	1 (0.4)	0 (0.0)
Programs of skills demonstration are flexible and adjustable for simulation class	Strongly disagree	5 (2.1)	17 (7.1)
	Disagree	16 (6.6)	27 (11.2)
	Neutral	71 (29.5)	16 (6.6)
	Agree	69 (28.6)	10 (4.1)
	Strongly agree	1 (4.1)	0 (0.0)
	Strongly disagree	41 (17.0)	12 (5.0)
During skills demonstration, the number of students per teaching group is small enough and appropriate for my learning	Disagree	117 (48.5)	26 (10.8)
	Neutral	9 93.7)	28 (11.6)
	Agree	1 (0.4)	4(1.7)
	Strongly agree	3 (1.2)	0 (0.0)
	Strongly disagree	22 (9.1)	28 (11.6)
During skills demonstration, I can get necessary help in the use of equipment	Disagree	4 (1.7)	6 (2.5)
	Neutral	102 (42.3)	19 (7.9)
	Agree	35 (14.5)	13 (5.4)
	Strongly agree	8 (3.3)	4 (1.7)
During skills demonstration, I can assess my own skills performance critically	Strongly disagree	13 (5.4)	21 (8.7)
	Disagree	36 (14.9)	28 (11.6)
	Neutral	72 (29.9)	14 (5.8)
	Agree	44 (18.3)	6 (2.5)
	Strongly agree	6 (2.5)	1 (0.4)
My teacher gives me necessary feedback related to my performance within a reasonable period of time	Strongly disagree	8 (3.3)	14 (5.8)
	Disagree	28 (11.6)	33 (13.7)
	Neutral	96 (39.8)	14 (5.8)
	Agree	37 (15.4)	9 (3.7)
	Strongly agree	2 (0.8)	0 (0.0)

### Factors associated with students’ satisfaction with simulation-based education

In bivariate logistic regression analysis, interest to study the midwifery profession, the accurateness of teachers’ information about the requirement of skills competence, perceived assistance during skills demonstration and practice, ‘the way my teacher taught the simulation is suitable to my learning style’, and skill practising programs per semester were statistically significant associated with the students’ satisfaction with simulation-based education.

Variables that showed statistically significant associations with the students’ satisfaction with simulation-based education in the bivariate analysis were entered into a multivariate logistic regression model to see the independent effect of each potential determinant while controlling for possible confounders.

After controlling the effect of other predictor variables, the multivariate logistic regression analysis showed statistically significant association between perceived assistance during skills demonstration and practice, ‘the way my teacher taught the simulation is suitable to my learning style’ and skill practising programs per semester and the students’ satisfaction with simulation-based education with p<0.05, as outlined in Table 2.

**Table 2:** Multivariate logistic regression of the factors associated with satisfactory simulation-based education among undergraduate midwifery students in public universities and colleges in Harar and Dire Dawa cities, Eastern Ethiopia, 2018

*Variables*	*Categories*	*Unsatisfied n (%)*	*Satisfied n (%)*	*AOR (95% CI)*	*p*
Perceived assistance during skills demonstration and practice	Not good	47 (19.5)	33 (13.7)	1	
	Good	23 (9.5)	138 (57.3)	5.622 (2.359–13.398)	0.000
The way my teacher taught the simulation is suitable to my learning style	No	63 (26.1)	39 (16.2)	1	
	Yes	7 (2.9)	132 (54.8)	22.391 (10.770–37.529)	0.023
Skills practising programs per semester	<2	44 (18.3)	41 (17.0)	1	
	≥2	26 (10.8)	130 (53.9)	2.344 (1.032–5.322)	0.042

AOR: adjusted odds ratio.

The study findings showed that those students who were assisted by their teachers during skills demonstration and practice were 5.6 times more satisfied than those who were not assisted (AOR=5.62; 95% CI: 2.36–13.40, p<0.001). The students who perceived that the way their teachers taught the skills demonstration was suitable to their learning styles were 22.4 times more satisfied with the simulation-based education (AOR=22.4; 95% CI: 10.8–37.5, p<0.023). Students who perceived that the number of skills practices per semester was enough were 2.3 times more likely to be satisfied with simulation-based education (AOR=2.3; 95% CI: 1.0–5.3, p<0.042).

## DISCUSSION

This study found that the proportion of satisfaction with simulation-based education and associated factors was 70.95% among the student population, which is lower than in studies conducted in other regions^13-15^. This difference might be due to differences in methodology, sociodemographic characteristics of participants and the technological gap between the countries. The proportion of student satisfaction found in this study with simulation-based education by their teachers, in skills learning and development of long-term skills, was higher than in a study conducted at the University of Gondar but similar to that in a study at King Saud bin Abdul-Aziz University for Health Sciences, Saudi Arabia^12,13^.

The efforts of teachers in assisting their students during skills demonstration, the suitability of the way of teaching to the learning styles of students, and the number of scheduled programs per semester for skills practising were statistically significant factors with satisfaction in simulation-based education. The proportion of student satisfaction with simulation-based education, found in the present study, is high compared to previous studies in Ethiopia. This study highlights the vital role of the effort of teachers’ assistance, the suitable way of teaching to learning styles, and the skills practising programs to student satisfaction, not only during simulation practice but also in actual patient care. The level of satisfaction of simulation-based learning among midwifery students in Ethiopia is low compared to those of recent studies conducted abroad. Therefore, proposing the means to get better teachers’ assistance, coordinating the teaching with the learning styles at simulation-based education and planning enough skills practising programs per semester are recommended. Creating the means to improve the overall quality of simulation-based education is thus advocated.

This study also showed that the availability of enough practising programs per semester was significantly associated with student satisfaction with simulation-based education.

### Limitations and strengths

The findings of this study suggest satisfaction with the learning method, but still this study has certain limitations. The study was conducted within public universities and colleges only. The study was carried out using a quantitative method only, hence we would recommend that further studies are needed using a qualitative methodological approach. Despite these limitations, the results of this study will help stakeholders to identify areas where students’ learning needs are not addressed, and accordingly help plan, develop and evaluate simulation-based learning in education in Ethiopia.

## CONCLUSIONS

Teaching institutions have to improve teachers’ skills to match the learning styles of their students. It may be necessary for teachers to have closer collaboration with their students, both during skills demonstration and skills practising in the clinical area, and to develop long-term pedagogical skills. Moreover, it is necessary to advocate that the curriculum designers and developers offer more simulation-based education and information regarding the necessity of skills competence for midwifery students in Ethiopia.
